# Management of Dermatologic Toxicities Associated With Targeted Therapy

**DOI:** 10.6004/jadpro.2016.7.3.18

**Published:** 2016-04-01

**Authors:** Mario E. Lacouture

**Affiliations:** Memorial Sloan Kettering Cancer Center, New York, New York

Oncologists who are already busy managing hematopoietic, gastrointestinal, and neurologic toxicities associated with cancer treatment must now learn to manage a consequence of new targeted agents—dermatologic side effects—according to Mario E. Lacouture, MD, Director of the Oncodermatology Program at Memorial Sloan Kettering Cancer Center, New York, New York.

The same pathways and proteins that are involved in malignant behavior are critical for the normal homeostasis and functioning of the skin. Therefore, oncologists are not only targeting cancer with monoclonal antibodies and small molecules, they are also targeting the skin, explained Dr. Lacouture.

At JADPRO Live at APSHO, Dr. Lacouture described the etiology and mechanism of action of dermatologic toxicity secondary to targeted therapies and discussed their evidence-based management.

The emergence of dermatologic toxicities is exacerbated by the fact that almost half of individuals diagnosed with cancer will have some skin condition before starting therapy. Commonly, they are tinea pedis/onychomycosis, xerosis, pruritus, and pyoderma—all of which can develop or become worse after receiving targeted therapies. According to Kilic, Gül, and Soylu (2007), about two-thirds of patients will receive chemotherapy, about half will receive radiotherapy, and most will have a surgical procedure. In almost half of these patients, these treatments invariably lead to or exacerbate skin toxicities, he indicated.

## NUMEROUS NEGATIVE CONSEQUENCES

Dermatologic conditions have a four-fold impact on psychological, physical, financial, and treatment outcomes. "They affect areas of the body that are exposed to other people," he said. "Patients lose their sense of privacy every time they have an acneiform rash because people at work ask them what’s wrong and they have to reveal their diagnosis."

Many drugs needed to treat dermatologic toxicities are not covered by insurance or carry additional, significant co-pays. There is also the effect on physical health, including pain and itching, that can limit one’s ability to conduct daily activities and get a good night’s sleep, he said.

Most importantly, he emphasized, dermatologic side effects can lead to inconsistencies in dosing, i.e., treatment interruption or discontinuation.

## WHY BE CONCERNED?

Oncologists should develop a good understanding of these toxicities or consult with a dermatologist. "In the majority of cases in which the oncologist wants to interrupt therapy, after patients are evaluated by a dermatologist, they can continue their therapy," Dr. Lacouture said.

But few patients see dermatologists, and even if they are referred, they may not be promptly seen. The median time for an appointment for a changing mole (possible melanoma) is 38 days (Tsang & Resneck, 2006), he noted, commenting, "Cancer patients cannot wait this long. They need immediate evaluation."

In a German survey, clinicians unaccustomed to seeing these toxicities typically graded them more severely (Hassel, Kripp, Al-Batran, & Hofheinz, 2010), and were more likely to delay treatment because of them (14%), than were dermatologists (7%). "Interruptions will rarely be needed due to a dermatologic event if oncologists familiarize themselves with these toxicities," added Dr. Lacouture.

Finally, a study that administered a quality-of-life questionnaire to 283 patients on cytotoxic or targeted therapies found that those treated with targeted therapies felt a greater impact on quality of life, especially in emotional domains (Rosen et al., 2013). "In other words," said Dr. Lacouture, "the emotions of your patients are going to be much more negatively impacted when you use targeted therapies that lead to skin toxicities."

## ACNEIFORM RASH: BIGGEST OFFENDERS

Dr. Lacouture provided an idea of when to expect rash with the various targeted agents:

*EGFR inhibitors* (panitumumab [Vectibix], afatinib [Gilotrif], erlotinib, cetuximab [Erbitux]): Between 75% and 90% of patients will develop an acneiform rash, which appears explosively within the first 2 to 4 weeks. Pruritus and tenderness also occur in 62% of patients (Drucker, Wu, Dang, & Lacouture, 2012).

*MEK inhibitors* (trametinib [Mekinist], cobemetinib [Cotellic]): An acneiform rash is seen in approximately 60% of patients (Flaherty et al., 2012). In melanoma, the combination of a BRAF inhibitor plus MEK inhibitor produces less skin toxicity vs. that produced by single agents.

*Anti-HER2 agents* (lapatinib [Tykerb], trastuzumab [Herceptin], etc.): Some of the anti-HER2 agents used in breast cancer can result in acneiform rash. Lapatinib is associated with rash in 50% of patients, but it is not severe (Rosen, Wu, Damse, Sherman, & Lacouture, 2012). Rash is rare with trastuzumab, but it can occur.

## RECOMMENDED PROPHYLAXIS AND TREATMENT OF RASH

Clinicians should follow a protocol for prophylaxis and treatment of acneiform rash (Table).

**Table:**
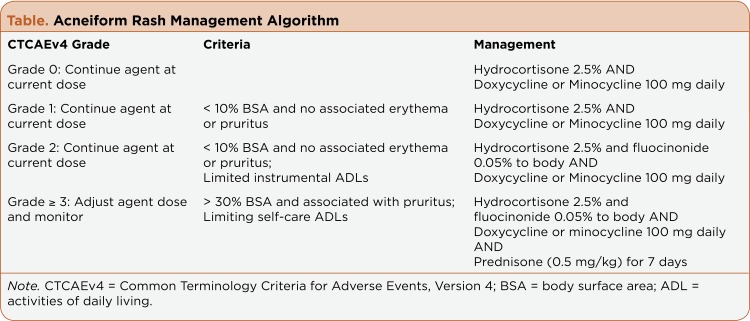
Acneiform Rash Management Algorithm

Oral antibiotics with topical steroids are recommended based on results of the phase 2 STEPP trial, which compared preemptive vs. reactive skin toxicity treatment in metastatic colorectal cancer (Lacouture et al., 2010). Prophylaxis reduced the incidence of grade 2 or worse skin toxicity by more than 50% and also reduced nondermatologic toxicities, including diarrhea, neutropenia, and dehydration, "probably because of a better maintenance of the barrier of the skin," Dr. Lacouture proposed.

In Dr. Lacouture’s opinion, antibiotics are a critical part of the armamentarium because EGFR inhibitors and MEK inhibitors cause immunosuppression in the skin. "These people are like children with eczema," he said. "Their skin becomes very easily infected."

He strongly encouraged clinicians to do bacterial culture swabs of skin discharge. "You will be surprised by the number of bacteria that are either gram-negative or resistant to your conventional therapies," he commented.

## RAF/BRAF INHIBITORS

Approximately 50% of patients taking RAF/BRAF inhibitors will develop maculopapular rash, which consists of macules and papules, flat red areas or elevated areas, and no puss bumps on the trunk (Boers-Doets et al., 2012). Topical corticosteroids are advised for grade 1 rash, and oral corticosteroids for grade 2/3 rash.

These drugs can also cause thickening of the skin, including painful calluses on the palms and soles (i.e., hand-foot syndrome). Prophylactic use of moisturizers that exfoliate the outermost layer of the skin can help, and prescription medications such as topical lidocaine or clobetasol can be used in severe cases.

## ADDITIONAL SIDE EFFECTS

*Dry skin*: 10% to 40% of patients treated with targeted agents experience severely dry skin and can develop painful fissures in the hands.

*Pruritus*: Pruritus is common with all of these agents but especially with EGFR inhibitors and checkpoint inhibitors. The anti-emetic aprepitant has been shown to reduce the severity of pruritus almost eight-fold (Santini et al., 2012).

*Brittle nails*: Poly-ureaurethane (Nuvail) and hydrosoluble nail lacquer (Genadur) have been FDA-approved to treat brittle nails. Biotin also induces more rapid and thicker growth of the nails.

*Paronychia*: 15% to 20% of patients treated with EGFR inhibitors develop a painful paronychia that can become secondarily infected. Nail discharge should be cultured. Partial nail avulsion may be required.

*Nonmelanoma skin cancer*: BRAF inhibitors are associated with skin cancer in about 20% of patients. Lesions, which tend to appear early in treatment, should be excited.

*Photosensitivity*: 40% of patients taking vemurafenib (Zelboraf) and vandetanib (Caprelsa) develop severe photosensitivity (and blisters), often within 10 minutes of sun exposure (Caro-Gutiérrez, Floristán Muruzábal, Gómez de la Fuente, Franco, & López Estebaranz, 2014). Sun protection is mandatory.

*Hair loss*: Targeted therapies (along with endocrine and cytotoxic agents) cause alopecia or abnormal hair growth in some patients. In patients undergoing chemotherapy, scalp cooling systems may help prevent these conditions.

## FINAL THOUGHTS

In conclusion, Dr. Lacouture emphasized, "Targeted therapies have an effect on skin, hair, and nails. The dermatologic care of cancer patients is very important in addition to everything else you do…Your patients are living longer, and these quality-of-life concerns are important to them."

## References

[1] Boers-Doets Christine B, Epstein Joel B, Raber-Durlacher Judith E, Ouwerkerk Jan, Logan Richard M, Brakenhoff Jan A, Lacouture Mario E, Gelderblom Hans (2012). Oral adverse events associated with tyrosine kinase and mammalian target of rapamycin inhibitors in renal cell carcinoma: a structured literature review.. *The oncologist*.

[2] Caro-Gutiérrez Dolores, Floristán Muruzábal Maria Uxúa, Gómez de la Fuente Enrique, Franco Ana Pampín, López Estebaranz José Luis (2014). Photo-induced erythema multiforme associated with vandetanib administration.. *Journal of the American Academy of Dermatology*.

[3] Drucker Aaron M, Wu Shenhong, Dang Chau T, Lacouture Mario E (2012). Risk of rash with the anti-HER2 dimerization antibody pertuzumab: a meta-analysis.. *Breast cancer research and treatment*.

[4] Flaherty Keith T, Robert Caroline, Hersey Peter, Nathan Paul, Garbe Claus, Milhem Mohammed, Demidov Lev V, Hassel Jessica C, Rutkowski Piotr, Mohr Peter, Dummer Reinhard, Trefzer Uwe, Larkin James M G, Utikal Jochen, Dreno Brigitte, Nyakas Marta, Middleton Mark R, Becker Jürgen C, Casey Michelle, Sherman Laurie J, Wu Frank S, Ouellet Daniele, Martin Anne-Marie, Patel Kiran, Schadendorf Dirk (2012). Improved survival with MEK inhibition in BRAF-mutated melanoma.. *The New England journal of medicine*.

[5] Hassel Jessica C, Kripp Melanie, Al-Batran Salah, Hofheinz Ralf-Dieter (2010). Treatment of epidermal growth factor receptor antagonist-induced skin rash: results of a survey among German oncologists.. *Onkologie*.

[6] Kiliç Arzu, Gül Ulker, Soylu Seçil (2007). Skin findings in internal malignant diseases.. *International journal of dermatology*.

[7] Lacouture Mario E, Mitchell Edith P, Piperdi Bilal, Pillai Madhavan V, Shearer Heather, Iannotti Nicholas, Xu Feng, Yassine Mohamed (2010). Skin toxicity evaluation protocol with panitumumab (STEPP), a phase II, open-label, randomized trial evaluating the impact of a pre-Emptive Skin treatment regimen on skin toxicities and quality of life in patients with metastatic colorectal cancer.. *Journal of clinical oncology : official journal of the American Society of Clinical Oncology*.

[8] Rosen Alyx C, Case Emily C, Dusza Stephen W, Balagula Yevgeniy, Gordon Jennifer, West Dennis P, Lacouture Mario E (2013). Impact of dermatologic adverse events on quality of life in 283 cancer patients: a questionnaire study in a dermatology referral clinic.. *American journal of clinical dermatology*.

[9] Rosen Alyx C, Wu Shenhong, Damse Amelia, Sherman Eric, Lacouture Mario E (2012). Risk of rash in cancer patients treated with vandetanib: systematic review and meta-analysis.. *The Journal of clinical endocrinology and metabolism*.

[10] Santini Daniele, Vincenzi Bruno, Guida Francesco M, Imperatori Marco, Schiavon Gaia, Venditti Olga, Frezza Anna M, Berti Pierpaolo, Tonini Giuseppe (2012). Aprepitant for management of severe pruritus related to biological cancer treatments: a pilot study.. *The Lancet. Oncology*.

[11] Tsang Matthew W, Resneck Jack S (2006). Even patients with changing moles face long dermatology appointment wait-times: a study of simulated patient calls to dermatologists.. *Journal of the American Academy of Dermatology*.

